# Ketogenic dietary interventions for autosomal-dominant polycystic kidney disease (ADPKD): a systematic review and synthesis without meta-analysis (SWiM) of observational and interventional studies^☆^

**DOI:** 10.1016/j.metop.2026.100447

**Published:** 2026-01-31

**Authors:** Maria G. Grammatikopoulou, Arriana Gkouvi, Kalliopi K. Gkouskou, Dimitrios Poulimeneas, Christina Tsigalou, Τheodoros Eleftheriadis, Odysseas Androutsos, Christos Cholevas, Ioannis Stefanidis, Maria Dalamaga, Dimitrios G. Goulis, Dimitrios P. Bogdanos

**Affiliations:** a Immunonutrition Unit, Department of Rheumatology and Clinical Immunology, Faculty of Medicine, School of Health Sciences, University of Thessaly, Biopolis, GR-41223, Larissa, Greece; b Department of Biology, Medical School, National and Kapodistrian University of Athens, Mikras Asias 75, GR-11527, Athens, Greece; c GENOSOPHY P.C., Athens, Greece; d Department of Nutritional Science and Dietetics, School of Health Sciences, University of the Peloponnese, GR-24100, Kalamata, Greece; e Department of Nutrition and Dietetics, School of Health Sciences and Education, Harokopio University, Athens, Greece; f Laboratory of Hygiene and Environmental Protection, Faculty of Medicine, Democritus University of Thrace, Alexandroupoli, Greece; g Department of Nephrology, Faculty of Medicine, School of Health Sciences, University of Thessaly, GR-41334, Larissa, Greece; h Lab of Clinical Nutrition and Dietetics, Department of Nutrition and Dietetics, School of Physical Education, Sport Sciences and Dietetics, University of Thessaly, GR-42132, Trikala, Greece; i Laboratory of Pharmaceutical Technology, Division of Pharmaceutical Technology, School of Pharmacy, Faculty of Health Sciences, Aristotle University of Thessaloniki, GR-54124, Thessaloniki, Greece; j Department of Biological Chemistry, Medical School, National and Kapodistrian University of Athens, 11527, Athens, Greece; k Unit of Reproductive Endocrinology, 1st Department of Obstetrics and Gynecology, Aristotle University of Thessaloniki, GR-54124, Thessaloniki, Greece

**Keywords:** Beta-hydroxybutyrate, Caloric restriction, Fasting, Ketone, Renal disease, Low carbohydrate, Ketogenic diet, Time-restricted feeding, High-fat diet

## Abstract

**Background/objectives:**

The purpose of this systematic review was to synthesize available human studies, present and weigh the evidence regarding the efficacy of ketogenic dietary interventions (KDIs) for ADPKD, and provide a direction for future research and relevant recommendations.

**Methods:**

Three databases were searched and risk of bias (RoB) of the studies was assessed using Cochrane's RoB 2.0, the Newcastle-Ottawa scale and the ROBINS-IΙ tool. The Synthesis Without Meta-analysis (SWiM) extension was used to present the results.

**Results:**

Eight studies were identified delivering interventions with ketogenic diets, intermittent fasting, time-restricted feeding, etc. KDIs were generally associated with weight loss and a more favorable anthropometric profile in most interventions (nine). Blood pressure remained unchanged in most interventions (five), similar to blood cholesterol, HDL-cholesterol, triglycerides and LDL concentrations. Regarding renal outcomes, eGFR was either higher (4 interventions) or remained stable post-intervention. htTKV remained unchanged in most of the studies. Evidence for renal structural change was inconsistent and limited by short intervention duration and small sample sizes.

**Conclusions:**

The evidence on KDIs for ADPKD is still limited. However, KDIs, particularly caloric-restriction diets, appear promising tools for managing ADPKD. Current human data support metabolic feasibility more consistently than renal disease modification. The effects of KDIs on renal structural outcomes remain uncertain and longer trials with appropriate comparators, namely the standard of care diet for ADPKD, are required before KDIs can be recommended for ADPKD.

## Highlights

• Ketogenic interventions can reduce body weight and improve the anthropometric profile of patients.
• Blood pressure, total cholesterol, HDL- and LDL-cholesterol, and triglycerides levels remain unaffected. Concerning renal outcomes, eGFR is either improved or remains stable post-intervention. htTKV also remains unchanged.
• Ketogenic interventions are feasible for ADPKD, however, research is still limited. More research is required in order to understand the potential of ketogenic diets to act as an adjuvant treatment for ADPKD.

## Graphical abstract

**Figure:**
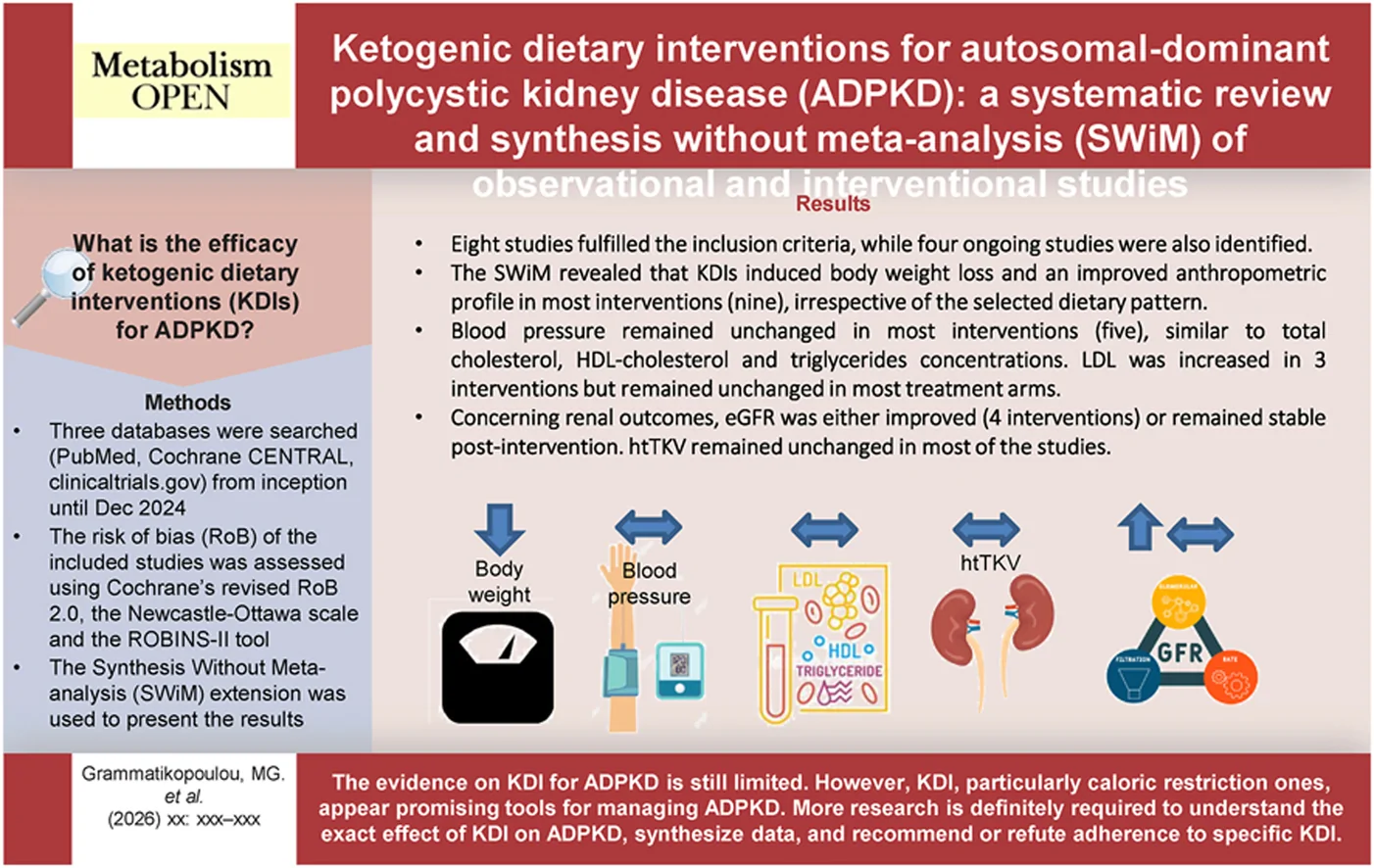

## 1 Introduction

Autosomal Dominant Polycystic Kidney Disease (ADPKD) is the most common inherited renal diagnosis leading to end-stage kidney disease (ESKD) [1] via the development and expansion of multiple cysts throughout the renal parenchyma. Although it is a rare disease, affecting one patient among 400 to 1000 live births [2], it accounts for 10% of the total cases of renal failure [3]. Therapeutic modalities for ADPKD involve symptom management (hypertension, infections, pain), halting disease progression (cyst development and growth), and dialysis in the final ESKD stages.

The pathogenesis of ADPKD involves a defective glucose metabolism via an extracellular signal-regulated kinase (ERK)-mediated pathway [4], indicating that affected renal epithelial cells rely heavily on glucose and aerobic glycolysis. According to Li [5], cystogenesis is driven by glucose transport into lumens of outwards-facing epithelia. Interestingly, pharmacological activation of AMP-activated protein kinase (AMPK) with metformin has been shown to be associated with delayed renal cyst growth in mouse models [6]. Tolvaptan also activates AMPK and has been shown to slow down cyst development. Patients with ADPKD and type 2 diabetes exhibit larger total kidney volume (TKV) than those with ADPKD alone [7], indicating a Warburg effect. As a result, Kipp et al. [8] suggested influencing renal mTOR activity using dietary manipulations as an alternative treatment for ADPKD. Notably, mTOR is highly sensitive to nutrient availability—particularly amino acids and glucose—as well as cellular energy status and growth factor signaling [8]. Glucose metabolism controls AMPK, whereas mTOR is regulated by various cues, including the availability of amino acids.

These mechanisms led to the recent research interest in ketogenic dietary interventions (KDIs) for ADPKD management. KDIs involve many approaches (oral nutrient supplements and complete dietary patterns) aiming to shift energy metabolism and provide alternate fuel, namely ketones. Ketogenic dietary patterns are diverse; however, their core recommendations are common, providing a low carbohydrate, high fat and moderate protein content [9]. On the other hand, oral nutrient supplements (ONS) used in KDIs consist mainly of medium-chain triglycerides (MCTs) or ketones (beta-hydroxybutyrate, BHB), provided with every meal, to assist ketosis [10]. KDIs include several variations of ketogenic diets (KDs), time-restricted feeding (TRF) or caloric restriction (CR) [11]. Regarding ADPKD, *post-hoc* analyses of the Developing Interventions to Halt Progression of ADPKD 1 (DIPAK 1) study revealed that every doubling in blood ketone (BHB) concentrations was associated with an improvement in the annual rate of eGFR by 0.33 mL/min/1.73 m^2^ [12]. However, patients with ADPKD adhere to the same diet as all patients with chronic kidney disease (CKD). This approach is because the recommendations for protein intake are strict for all patients with CKD [13], whereas, for most of the public, KDIs are falsely equivalent to a high-protein diet. In parallel, not all healthcare professionals are KDI-savvy, and not all patients can adhere to stricter dietary patterns like some ketogenic ones. As a result, research on the possible effectiveness of KDIs for ADPKD remains limited, although the approach is novel. For this, the present systematic review aimed to review and synthesize all available human studies and to present and weigh the evidence regarding the efficacy of KDIs for ADPKD to provide a direction for future research and relevant recommendations.

## 2 Materials and methods

### 2.1 Study protocol, PICOS and search strategy

**Fig. 1:**
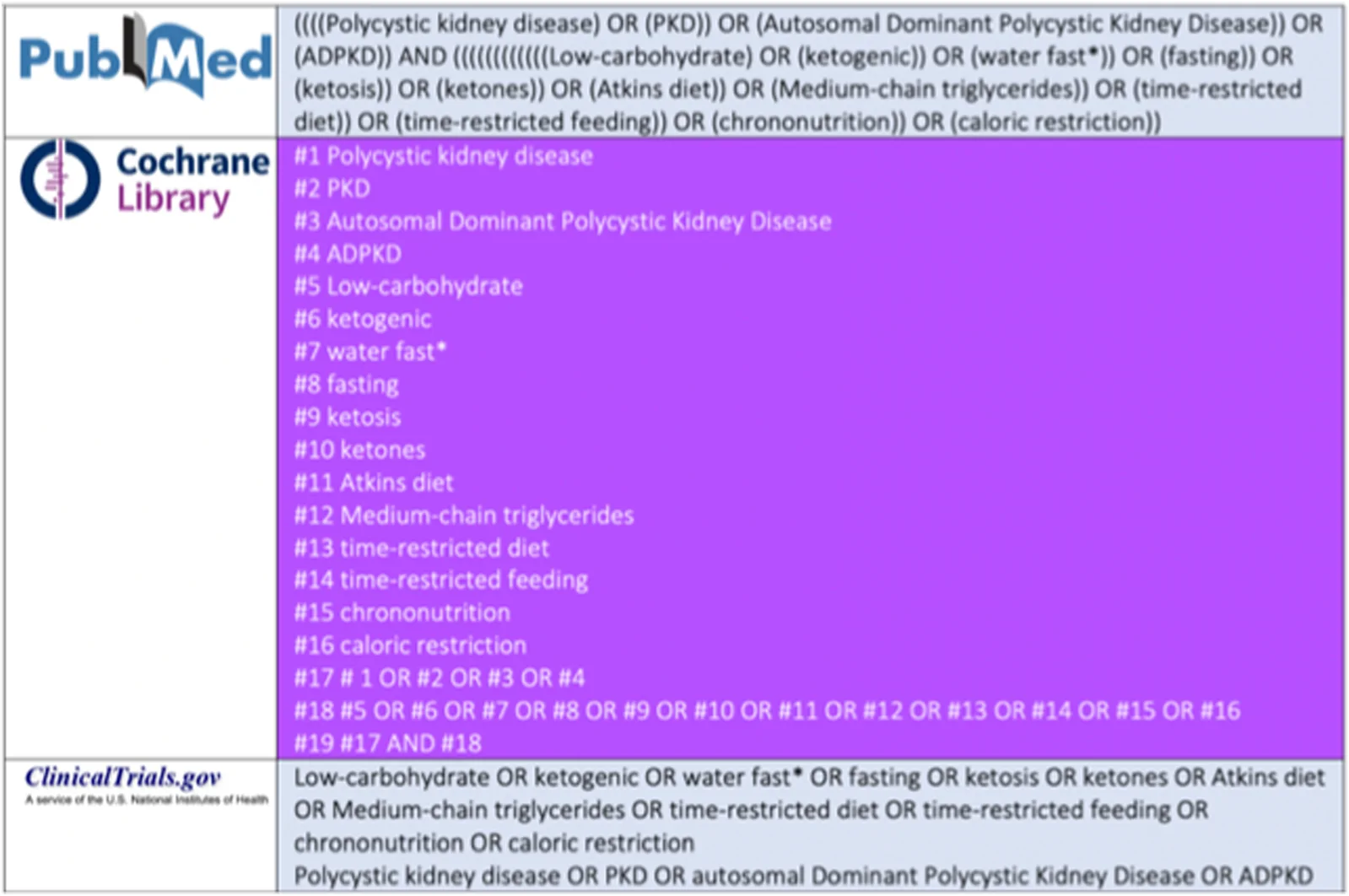
Search syntaxes used.

The Preferred Reporting Items for Systematic reviews and Meta-Analyses (PRISMA) [14] and the Synthesis Without Meta-analysis (SWiM) extension [15] were used for the presentation of the present review. The PICOS (Population, Intervention, Comparison, Outcomes, Study design) of the research question is presented in Supplementary Table 1. The research question was “For patients with ADPKD, what is the effect of KDI on PKD-related outcomes?” Three databases were searched for relevant studies: PubMed, the Cochrane Central Register of Controlled Trials (CENTRAL) and clinicaltrials.gov. Searches were also conducted in the gray literature and citations of the retrieved items. The search strategy for each database is presented in Fig. 1. The systematic review and synthesis without meta-analysis protocol was registered at the Open Science Framework (OSF) (https://shorturl.at/2W5LA).

### 2.2 Inclusion and exclusion criteria

Studies were included in the systematic review when (i) involving patients with ADPKD, (ii) examining the use of KDIs, specifically KD, TRF or CR, (iii) including any comparison, such as usual diet or other diets, or no comparison, (iv) having any intervention duration, (v) published until December 2024 (vi), adopting a cohort, case-control, unrandomized or randomized clinical trial design and, (vii) when written in the English language.

Studies were excluded when (i) pooling patients with ADPKD with other CKD diagnoses, (ii) involving animal or pre-clinical studies, (iii) examining non-KDIs, (iv) involving case reports, literature reviews, systematic reviews and meta-analyses, and (v) when published in another language.

### 2.3 Outcomes of interest

Outcomes of interest involved any outcome related to ADPKD, kidney health, the health of patients and KDI feasibility/adherence, including eGFR, TKV, arterial blood pressure (BP), lipid profile, quality of life (QoL), health assessment, etc.

### 2.4 Risk of bias

Two authors independently assessed eligible studies for bias using the appropriate tool for each study design. Cochrane's revised Risk of Bias (RoB) tool 2.0 [16] was applied to randomized controlled trials (RCT). Judgments were made if there was a low risk, some concerns or high risk of bias regarding the randomization process, deviations from intended interventions, missing outcome data, measurement of the outcomes, selection of the reported results and the final assessment regarding the overall bias. For cohorts, the Newcastle-Ottawa scale (NOS) [17] was applied, and the maximum score for each study was 9 points. Finally, for non-randomized interventions, the Risk Of Bias In Non-randomized Studies - of Interventions (ROBINS-I V2) tool was employed [18] to assess bias.

### 2.5 Data extraction

Two independent researchers extracted data in predefined Excel spreadsheets. Information regarding the sample, recruitment, country of origin, funding, design and methodology (randomization particularities, masking), intervention (KDI particularities) and comparator arms, outcomes of interest, dropouts, adverse events, presented analysis, and general results were extracted for all studies.

### 2.6 Data synthesis

Due to the expected high heterogeneity of the study designs and KDIs applied, a meta-analysis was not deemed safe. Instead, a SWiM was performed using vote counting based on the direction of effect (mean differences) for each outcome [19] to accompany the narrative synthesis [20]. The KDI characteristics of each study and the design were used to assess heterogeneity, according to the Cochrane Handbook [20] and the SWiM guidelines [15].

## 3 Results

### 3.1 Studies fulfilling the inclusion criteria

**Fig. 2:**
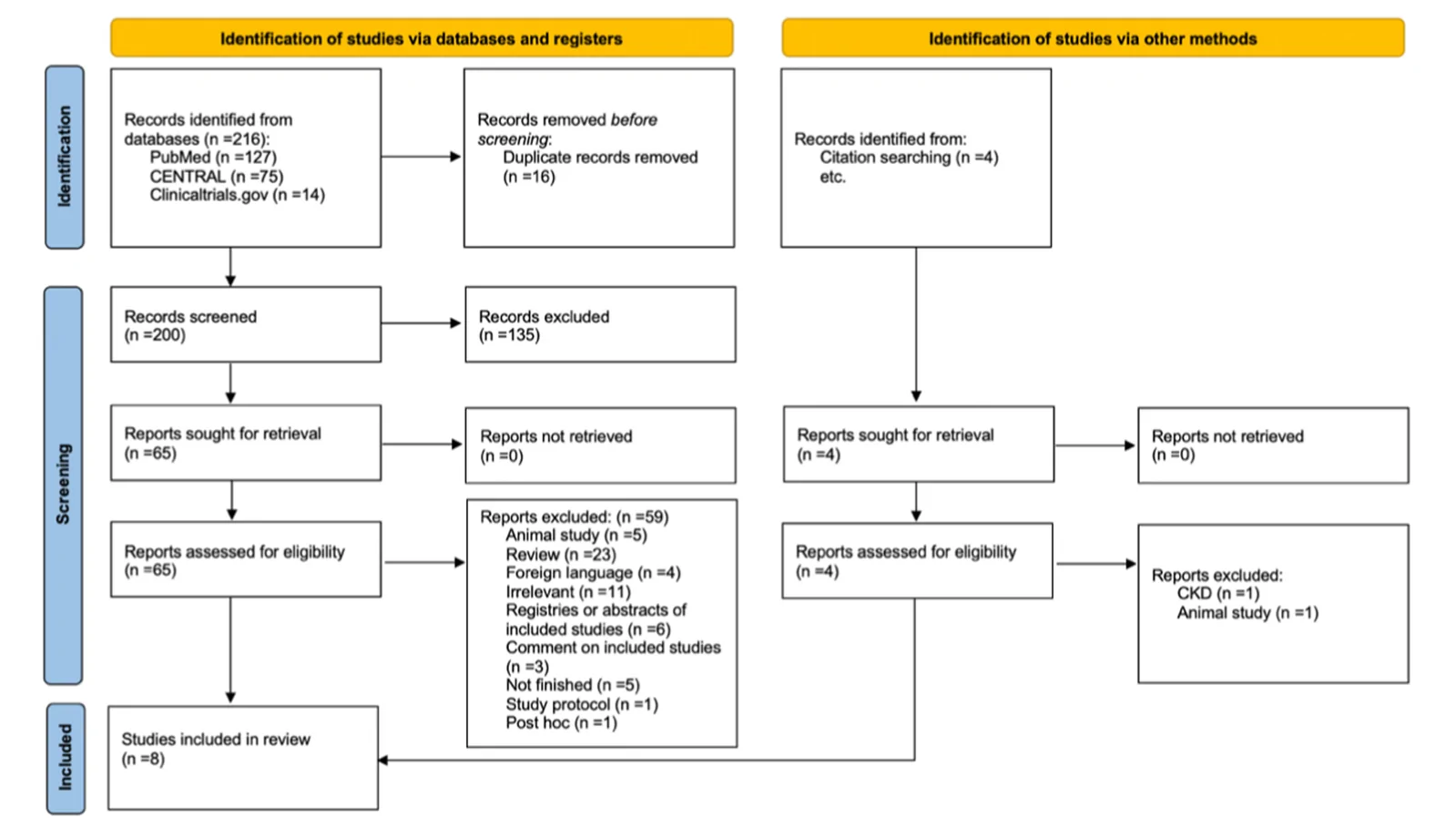
The PRISMA 2020 flowchart of the studies selection process.

Fig. 2 showcases the PRISMA flowchart, presenting the studies’ selection process. Excluded studies are detailed in Supplementary Table 2. Of the 216 studies screened, 16 duplicates were removed, and 200 articles were screened at the title and abstract level. Of these, 65 studies were assessed at a full-text level. Eight studies in total [three RCTs [[21], [22], [23], [24], [25]], one proof-of-principle non-randomized controlled trial [26], one uncontrolled trial [27], one retrospective [11] and two prospective cohorts [28,29] were performed on humans and fulfilled the study criteria. The characteristics of these studies are detailed in Table 1. All research items were retrieved in full-text format. The studies were performed in Germany [11,21,22,26]**,** the USA [[23], [24], [25],^28^], Italy [30] and Turkey [29]. Additionally, four unpublished study protocols were retrieved on clinicaltrials.gov.

**Table 1:** Characteristics of the interventional studies investigating ketogenic therapies on patients with ADPKD.

First author	Bruen [28]	Cukoski [21,22]	Ekinci [29]	Hopp [23]	Oehm [26]	Steele [24,25]	Strubl [11]	Testa [30]
**Study name**	Ren.Nu	KETO-ADPKD	–	–	RESET-PKD	–	Experience of people with ADPKD with KDs	GREASE
**Publication**	*Kidney Dial.* 2022	*Cell Rep Med*. 2023; *Nephrol Dial Transplant*. 2024	*Clin Nephrol. 2018*	*iScience.* 2022	*Nephrol Dial Transplant*. 2023	*J Am Soc Nephrol.* 2023; *Clin Kidney J.* 2025	*Clin Kidney J*. 2021	*Pharma Nutr.* 2019
**Publication form**	Full-text	Full-text and abstract	Full-text	Full-text	Full-text	Abstract, Full-text	Full-text	Full-text
**Origin**	USA	Germany	Turkey	USA	Germany	USA	Germany	Italy
**Registration**	NR	NCT04680780	NR	NCT03342742	NCT04472624	NCT04534985	NR	
**Funding**	RenAlign, Santa Barbara Nutrients Inc.	PKD Fndn, Marga and Walter Boll-Stiftung, German Research Fndn, Koeln Fortune Program, Fritz-Scheler-Scholarship	Scientific Research Projects Unit of Bezmialem Vakif University	NIH NIDDK, Colorado Clinical and Translational Sciences Institute, NIH NCATS Colorado Clinical and Translational Science Award, PKD Fndn, University of Colorado Nutrition Obesity Research Center	Koeln Fortune Program, Ministry of Science North Rhine-Westphalia, German Research Fndn	NIH NIDDK, Zell Family Fndn	Amy P. Goldman Fndn, Jarrett Family Fund, Deutsche Forschungs-gemeinschaft, Koeln Fortune Program, Ministry of Science Northrhine-Westfalia, PKD Fndn, Thermo Fisher Scientific, Fresenius Kabi, Otsuka Pharmaceuticals	Menarini Diagnostics
**Design**	Prospective cohort	RCT	Prospective cohort	RCT	Proof-of-principle, non-RCT	RCT	Retrospective cohort	Uncontrolled clinical trial
**Blinding**	Open-label	Single-blind (MRI readers)	Open-label	Single blind	Open-label	Single-blind (investigators and outcomes assessors) *	Open-label	Open-label
**Randomization**	N/A	On 1:1:1 using random permuted blocks size 3	N/A	1:1 with a blocked sequence with sex stratification	N/A	NR	N/A	N/A
**Participants (N)**	N = 24 patients with ADPKD (eGFR ≥30 mL/min/1.73 m^2^), not on tolvaptan	N = 66 patients with ADPKD (Mayo Class 1A–1E, eGFR 84.01 mL/min/1.73 m^2^)	N = 65 with ADPKD who had normal to near-normal eGFR values	N = 28 patients with overweight/obesity and ADPKD (eGFR ≥30 mL/min/1.73 m^2^), not on tolvaptan	N = 10 patients with rapidly progressed ADPKD (Mayo Class 1C–1E, eGFR ≥45 mL/min/1.73 m^2^)	N = 29 patients with ADPKD	N = 131 patients with ADPKD on KDI	N = 3 patients with ADPKD with eGFR 45–89 mL/min/1.73 m^2^, not on tolvaptan
**Recruitment**	Social media or personal invitations	University Hospital Cologne	Nephrology Unit of Bezmialem Vakif University Medical Faculty	University of Colorado Anschutz Medical Campus	German AD(H)PKD cohort (NCT02497521) or through the patients' advocacy organization “Familiäre Zystennieren e.V.”	Recruited nationally NOD	Social media groups and patient advocacy groups in the USA and Germany	Azienda Ospedaliero-Universitaria di Modena
**Pre-intervention**	–	Usual CHO-rich diet	NR	NR	High-CHO diet for 2–4 wks	–	–	–
**Intervention**	Remote PBKD training program and intervention paired with TRF (*n* = 20)	KD (*n* = 23)	TRF during Ramadan (*n* = 24)	IMF with CR (*n* = 11)	KD (*n* = 5) for 14 days	TRF (8h window) (*n* = 14)	KD (*n* = 74)	MAD (*n* = 3)
**Comparator A**	–	WF (*n* = 22) for 3 consecutive days within the first 14 days of each month and *ad libitum* intake for the rest of the month	No intervention (*n* = 41)	CR (*n* = 13)	WF (*n* = 5) for 3 days	Healthy eating recommendations (*n* = 15)	TRF (*n* = 52), mostly involving the 16:8 regimen (8h eating window each day)	–
**Comparator B**	–	No intervention (*n* = 21)	–	–	–	–	CR (*n* = 5) NOD	–
**Intervention Duration**	5 months approximately	3 months	1 month	12 months (3 months intensive, 9 months maintenance)	14 days or 3 days, respectively	12 months	6 months approximately	3 months
**Post-intervention**	–	Return to normal diet	NR	NR	Normal, *ad libitum* diet (CHO-rich) for 3–6 weeks	NR	NR	–
**KDI type(s)**	The PBKD was low-CHO/high-fat, alkaline diet (PRO intake ≤0.8 g/kg) with some dairy, eggs, and fish. TRF was also recommended to raise ketones and lower FBG/Ins. Renal stressors (oxalate, inorganic phosphate, purines/UA) were reduced to avoid microcrystal renal injury. Nutrient-dense, whole foods were chosen and processed foods were minimal. Materials on the PBKD, recipes, videos, and guides were provided. KetoCitra (ready-to-mix powder) was consumed twice/d with meals. Private social media groups provided peer and health support. Patients met one-on-one, virtually, with an RDN thrice. Information regarding the TRF advice was not provided.	Isocaloric, classical KD, with <30 g CHO/d and a moderate-to-low protein intake (0.8 g/kg BW). Offered for omnivores and vegetarians, with shopping lists, recipes, protein bars (Adonis Smart Foods) and diet support. Upper intake limits were set for oxalate (100 mg/d), NaCl (7 g/d), P (700 mg/d) and K (4000 mg/d). Patients were encouraged to limit meat intake.	TRF for approximately 17 h/d, fasting for >23/30 days during Ramadan, who also fasted the whole week before the end of Ramadan.	Both groups reduced TEI by 34%/week on a 55% CHO, 15% protein, 30% fat diet. The CR arm had a 34% TEI deficit with individual weight maintenance goals (REE x activity factor of 1.5). In CR, specific support strategies included counting calories, portion size, and daily food logging. The IMF arm reduced TEI (20% EER) as a single meal on 3 non-consecutive days/wk, resulting in a weekly energy deficit of 34% (similar to the CR). Sample fast-day menus and individualized fast-day energy goals were given. On fast days, patients consumed their calories at their meal of choice. On fed days, IMF patients ate *ad libitum*, but with healthy food and portion choices.	Isocaloric, classical KD, based on a fat: PRO:CHO ratio of 10:4:1. Of the fat calories, 10% were provided as MCT oil with ketogenic snacks.	TRF within an 8-h window, beginning within 3 h of waking. Moderate dietary Na restriction (2.3–3 g), appropriate hydration, protein intake of 0.8–1.0 g/kg of ideal BW, moderate daily phosphate restriction (800 mg), Na restriction (2.3–3 g), and moderation in caloric intake (no target).	Different KDI	MAD with 20 g of CHO (about 5% of TEI), PRO 30% and fat 65% of TEI. A sugar-free MV ONS was provided to correct micronutrient deficiencies. Personalized recipes and instructions for creating variations to recipes were provided.
**WF particularities**	N/A	Consume only non-caloric drinks (e.g., water, tea or coffee without milk) and a broth once daily	NR	N/A	Limited oral intake to *ad libitum* amount of water and a low-salt broth once a day	N/A	N/A	N/A
**Dropouts**	*n* = 2 for unknown reasons and *n* = 2 did not complete the final visit assessment	*n* = 3 patients never initiated the trial (2 controls and 1 in the WF arm), *n=2* patients in the WF arm dropped out (one due to feasibility and one due to personal reasons)	*n=1* from TRF and *n* = 9 from the control group were lost to follow-up, *n=1* from the control group got pregnant, *n=2* from TRF stopped at 1 week due to AKI	*n* = 2 in the CR and *n* = 1 (IMF) for reasons unrelated to tolerability and *n* = 1 (IMF) stopped before month 3 due to diet tolerability. *n* = 2 (CR) discontinued the intervention before 12 months but stayed in the analysis for data until month 3	None	*n=6*	N/A	none
**Primary outcomes**	Tolerability, feasibility, adherence, BP, BW, FPG, serum CREAT, eGFR, BUN, lipid profile (TG, LDLc, HDLc, TC), ALB	Objective adherence and patient-reported feasibility (questionnaire)	BP, BW, eGFR, serum CREAT, BUN, UA, ALB, FPG, lipid panel, electrolytes, 24h urine PROT, 24h urine volume, NGAL, KIM-1	Feasibility, BW loss, htTKV (MRI)	Renal and liver volumetry (MRI), ketone bodies (Ac and BHB), BMI, waist perimeter, BP, body composition (BIA)	Tolerability, feasibility, adherence, BP, BW, FPG, eGFR, lipid profile (TG, LDLc, HDLc, TC), CRP, htTKV (MRI)	BP, eGFR, BMI, health improvements, AEs, feasibility	MAD tolerability
**Secondary outcomes**	Wellness, dietary satisfaction, overall satisfaction	Renal and liver volumetry (MRI), IGF-1, hsCRP, BP, eGFR, urinary A1M, albuminuria	–	BP, anthropometry, eGFR, lipid panel, CRP, HbA1c, TSH, QoL, mood (POMS2), binge eating (QEWP-5), PA, IL-6, IL-18, HOMA-IR, IGF-1, IGFBP-1, adiponectin, leptin, ghrelin, and PBMC markers	(not separated from primary)	BW loss, abdominal adiposity, IGF-1, PBMC, IGFBP-1, BHB, QoL, mood (POMS2)	–	Δ in BW, eGFR, glycemia, BP, CREAT, ketonemia (BHB), proteinuria, TC, LDLc, HDLc, TG
**Other outcomes**	–	ECG, BW, body composition, BHB		SAT, VAT, TAT	–	–	–	–
**Timepoints**	Monitoring and logging dietary intake, ketone concentratioms, BP, BW, urinary pH, and blood sugar were encouraged but voluntary. Metabolic panel was obtained at baseline and post-program	Baseline and at the end of each month	Baseline, 1-month AR. For the intervention group, also ER	Baseline and at each month	Baseline and at the end of each phase (V1, V2, V3, V4)	NR		Baseline and at the end of each month
**Analysis**	PP	PP	PP	ITT	ITT	ITT		none
**Ketosis assessment**	fingertip blood and urine pH	Two daily at-home Ac breath analyses and blood BHB	NR	NR	Breath (portable breath analyzer), urine and fingertip blood, all recorded in a diary	BHB	Self-reported ketone body concentrations in urine, blood or breath	Twice a day by finger puncture and a portable blood analyzer
**Diet record**	Using the Cronometer smartphone app	None; adherence was based on ketone assays	None	Diet records and self-reported dietary adherence	Diet diary	Meal logging, 7-d photographic food records		Through weekly meetings with dietitians
**AEs**	One UTI, one gout flare, and one passed renal stone	Mild flu-like symptoms in the KD, hyperlipidemia, elevated UA, renal stones	2 patients developed AKI and discontinued fasting. They recovered after a few days.	Hunger, GI distress, fatigue, cold intolerance, irritability, insomnia, mood change, etc.	Hunger (WF), self-limited palpitations (*n* = 2), elevated UA (KD, WF), cholesterol and LDLc (KD) and serum bilirubin (WF)	NR	66% reported AEs, frequently observed with KDI. The most common concern was hyperlipidemia. One patient experienced kidney stones and two increases in CREAT.	Fatigue (*n* = 1), muscle cramps (*n* = 1), increase in TC concentrations.
**Results**	All patients reached ketosis. Most (89%) reported BW loss; in 83%, BP improved (many patients lowered BP medication dosages). Serum CREAT trended downwards (−5.8%), whereas eGFR showed an average increase of 8.6% (4.4 mL/min/1.73 m^2^) from baseline. BUN trended downwards (−10.1%) and FBG levels decreased (−16.5%). Electrolytes were within normal ranges except for *n* = 1 with low Na (129 mM) post-intervention, with no hyponatremia signs. Lipid profiles trended towards improvement, including TG and HDLc. Averages of TC, LDLc, and ALB levels remained similar.	Both interventions induced ketogenesis (blood and breath Ac). In the KD, 95% of patients reported the diets as feasible compared to 85% following the WF. The KD reduced BF and TLV, and TKV, the latter, non-significant. Overall, the KD improved renal function, while the control and WF groups showed a progressive decline, as typically in ADPKD.	No changes occurred in either group in BP, BW, CREAT, 24-h urine volume, NGAL, KIM-1, or eGFR, while 24-h urinary PRO was decreased in TRF.	Clinically significant BW loss occurred in both interventions. BW loss was greater and adherence and tolerability were better on the CR arm. Slowed renal growth correlated with BW and VAT loss, independent of dietary regimen.	Ac breath and BHB blood concentrations increased in both arms. Nine of 10 patients reached a ketosis state and 9/10 evaluated KDI as feasible. TKV did not change during the intervention, but a significant impact on ΔTLV was noted (V2 to V3: −7.7%), mediated by changes in its non-cystic fraction.	BW was modestly reduced in both groups, and the rate of htTKV increase was lower in TRE, which correlated with BW and VAT loss. The 8-h window was difficult to adhere to.	Most patients (80%) reported that KDI improved their overall health, 67% described improvements in ADPKD-related health, 90% observed BW loss, 64% of those with hypertension noted BP improvements, 66% noticed AEs frequently observed with KDI, 22 reported safety concerns (hyperlipidemia), 45 patients reported small improvements in eGFR and 92% reported KDI as feasible, with 53% incorporating breaks during their diet. The KD cohort reported a more profound effect than the TRF one.	Patient satisfaction and compliance with the diet were high. Wellness reached the highest score, whereas BW and glycemia were lowered.

Δ: change; A1M: Alpha-1-microglobulin; Ac: acetone; AEs: adverse events; ADPKD: autosomal-dominant polycystic kidney disease; AKI: Acute Kidney Injury; ALB: albumin; AR: After Ramadan; BIA: bioelectrical impedance analysis; BF: body fat; BHB: beta-hydroxybutyrate; BMI: body mass index; BP: blood pressure; BUN: blood urea nitrogen; BW: body weight; CHO: carbohydrate; CR: caloric restriction; CREAT: Creatinine; d: day; ECG: electrocardiogram; EER: Energy expenditure requirements; eGFR: estimated glomerular filtration rate; Fndn: foundation; FPG: fasting blood glucose; HbA_1c_: glycated haemoglobin; HDLc: high-density lipoprotein cholesterol; HOMA-IR: Homeostatic Model Assessment for Insulin Resistance; hsCRP: high-sensitivity C-reactive protein; htTKV: height adjusted TKV; IGF-1: insulin-growth factor 1; IMF: Intermittent fasting; IL-6: interleukin 6; IL-18: interleukin 18; Ins: Insulin; ITT: intention-to-treat; K: Potassium; KD: ketogenic diet; KDI: ketogenic dietary interventions; KIM-1: kidney injury molecule-1; LDLc: low-density lipoprotein cholesterol; MAD: modified Atkins diet; MCT: medium-chain triglycerides; MRI: magnetic resonance imaging; MV: multivitamin; Na: Sodium; Na: Sodium; N/A: not applicable; NGAL: neutrophil gelatinase-associated lipocalin; NOD: not other defined; NR: not reported; ONS: oral nutrient supplement; P: Potassium; PA: physical activity; PBKD: plant-based ketogenic diet; PBMC: peripheral blood mononuclear cell; POMS2: Profile of Mood States 2; PP: per protocol; PRO: protein; QEWP-5: Questionnaire on Eating and Weight Patterns-Revised; QoL: quality of life; RCT: Randomized controlled trial; RDN: registered dietitian-nutritionist; REE, resting energy expenditure; SAT: subcutaneous adipose tissue; TAT: total adipose tissue; TC: total cholesterol; TEI: Total energy intake; TG: triglycerides; TKV: total kidney volume; TLV: total liver volume; TRF: time-restricted feeding; UA: Uric acid; USA: United States of America; UTI: Urinary tract infection; VAT: visceral adipose tissue; WF: water fasting, wk: week; * Abstract and full text indicates single-blind, registry reports double-blind (patients unblinded).

The sample size ranged from three [30] to 131 patients with ADPKD [11]. Patients were aware of the intervention in all eligible research, and in three studies, investigators or outcomes assessors were blinded (single-blind) [[21], [22], [23], [24], [25]]. The inclusion and exclusion criteria of each study are listed in Supplementary Table 3. As shown in Table 1, most studies were short in duration and the comparators varied substantially; notably only one RCT directly compared a KDI approach (TFR) against typical healthy eating recommendations for ADPKD [24,25].

### 3.2 KDIs

Performed KDIs varied widely, including KDs, TRF, CR, or a combination of these patterns. The Ren.Nu. study offered a low-carbohydrate, high-fat KD paired with TRF to increase ketosis [28]. In the KETO-ADPKD trial [21,22] and the RESET-PKD [26] cohort, a classical KD was compared to water fasting (WF). Hopp et al. [23] offered a combined intermittent fasting (IMF)-CR intervention (low-calorie IMF), compared to CR alone. In a randomized manner, Steele [24,25] compared TRF (8-h eating window) to the typical healthy eating recommendations for ADPKD. Strubl [11] used participants who were either on KD or TRF, whereas, in the GREASE [30] trial, the modified Atkins diet (MAD) pattern was applied without a comparator arm. Finally, Ekinci et al. [29] utilized the TRF for approximately 17 h of fasting during Ramadan. As described in Table 1, KDIs included different exposures (eg isocaloric carbohydrate restriction, explicit caloric restriction, time restriction, or short water fasts) with variable behavioral support intensity and ketosis monitoring.

### 3.3 Ongoing research

Table 2 details the studies that are still ongoing, without any published results to date, registered on clinicaltrials.gov. Three [[31], [32], [33], [34]] consist of parallel RCTs, with the interventions known to the patients and the investigators being blinded. The last [35] consists of an open-label, single-group intervention study. GREASE II [31,32] forms the continuation of GREASE [30] and aims to assess the efficacy of MAD versus a balanced normocaloric diet for ADPKD. On the other hand, the Daily Caloric Restriction in ADPKD [33] study involves CR through a combined diet and exercise intervention versus standard care for ADPKD. Both trials focus on the induced changes in TKV alongside other outcomes, and results are awaited to better understand the efficacy of KDIs. The Daily Caloric Restriction in ADPKD [33] has already published numeric results in clinicaltrials.gov; however, a statistical analysis and journal publication are still awaited. The more recent Renal Oxygen Consumption, Insulin Sensitivity, and Daily Caloric Restriction in ADPKD (EXPLORE) [34] trial investigates CR versus standard care. It evaluates changes in renal O_2_ consumption and insulin sensitivity. Finally, the Well-Formulated Ketogenic Diet Polycystic Kidney Disease [35] trial assesses the effects of a strict KD (<50 g of CHO/daily) on the TKV, eGFR, microalbuminuria, arterial BP, and ketosis of patients with ADPKD.

**Table 2:** Ongoing trials examining KDI in patients with ADPKD.

Name	GREASE II [31,32]	Daily Caloric Restriction in ADPKD [33]	Well-Formulated Ketogenic Diet Polycystic Kidney Disease [35]	Renal Oxygen Consumption, Insulin Sensitivity, and Daily Caloric Restriction in ADPKD (EXPLORE) [34]
**CTI**		NCT04907799	NCT06325644	NCT06496542
**Origin**	Italy	USA	USA	USA
**Funding**	Italian Ministry of Health	University of Colorado, Denver	Ohio State University	University of Colorado, Denver
**Published protocol**	*PharmaNutr.* 2020; *Nephrol Dial Transplant*. 2023	Clinicaltrials.gov	Clinicaltrials.gov	Clinicaltrials.gov
**Design**	Parallel RCT, single-blind (researchers)	Parallel RCT, double-blind (Investigator, Outcomes Assessor)	Open-label, single-group intervention	Parallel RCT, double-blind (Investigator, Outcomes Assessor)
**Participants**	N = 90 patients with ADPKD	N = 126 patients with ADPKD	N = 20 patients with ADPKD and deemed high risk for progression to ESKD	N = 20 patients with ADPKD who are overweight or obese
**Interventions**	a) KD (MAD, with 20 g CHO, PRO 25–30% of TEI, fat 60–70% of TEI, mainly PUFA and MCT) + K or Mg Citrate ONS + sugar-free MV ONS b) BND (CHO 50–60% of TEI, PRO 10–15% of TEI, fat 20–30%, 30 g fiber) + K or Mg Citrate ONS	a) daily CR (diet and exercise) b) standard care	KD (<50 g CHO, ∼1 g/kg of reference BW PRO)	a) CR (30% reduction in caloric intake and increased physical activity) b) standard care
**Intervention Duration**	12 months	24 months	52 weeks	2 years
**Endpoints**	TKV, tolerability and safety, eGFR, urinary β_2_MG and MCP-1	TKV, abdominal adiposity, adiponectin, leptin, IGF-1/IGFBP-1, TNF-α, IL-6, CRP, PBMC, gut microbiota, renal oxygen consumption, plasma metabolome	TKV, eGFR, microalbuminuria, BP, ketosis	Changes in renal O_2_ consumption, changes in insulin sensitivity
**Status**	Ongoing	Completed with preliminary numerical results published on clinicaltrials.gov	Ongoing	Ongoing

β_2_MG: Beta-2 Microglobulin; ADPKD, autosomal-dominant polycystic kidney disease; BND: balanced normocaloric diet; BW: Body weight; CHO: carbohydrate; CR: caloric restriction; CRP: c-reactive protein; ESKD: End stage kidney disease; IGFBP-1: Insulin-like growth factor-binding protein 1; IGF-1: insulin-like growth factor-1; IL-6: interleukin 6; K: Potassium; KD: Ketogenic diet; MAD: Modified Atkins diet; MCP-1: Monocyte chemoattractant protein-1; MCT: medium-chain triglycerides; Mg: Magnesium; MV: multivitamin; ONS: oral nutrient supplements; PBMC: peripheral blood mononuclear cell; PRO: protein; PUFA: poly-unsaturated fatty acids; RCT: randomized controlled trial; TEI: total energy intake; TKV: total kidney volume; TNF-a: Tumor necrosis factor a.

These ongoing trials are larger and longer than most published studies (12–24 months, including up to 126 participants) with standard of care as comparators. Clinically, these designs are more aligned with evaluating disease-modifying potential than short, feasibility focusesd studies and will be likely more informative for the development of future practice recommentations.

### 3.4 Studies outcomes

All studies evaluated the change in anthropometric parameters, including body weight (BW), body fat (%BW or fat mass), subcutaneous, visceral, and total adipose tissue (SAT, VAT and TAT, respectively), and the patients’ waist and hips circumferences.

Inflammation markers, mainly as secondary outcomes, were also selected, including interleukin (IL) 6 and 1, tumor-necrosis factor α (TNF-α), albumin, C-reactive protein (CRP), and high-sensitivity CRP (hs-CRP). Insulin-growth factor 1 (IGF-1) was also evaluated.

Ketosis was assessed through blood BHB or breath Acetone (Ac) concentrations. Other metabolism-related outcomes involved total cholesterol (TC), triglycerides (TG), high-density and low-density lipoprotein cholesterol (HDLc and LDLc, respectively), fasting plasma glucose (FPG), glycated hemoglobin (HbA_1c_), and apolipoprotein β (ApoB) concentrations. Arterial BP was also evaluated in most studies [11,[21], [22], [23],^26^,^28^,^29^].

Renal function-related outcomes used included the eGFR, blood-urea nitrogen (BUN) or urea, creatinine, α-1-microglobulin (A1M)-to-creatinine ratio, height-adjusted total kidney volume (htTKV), height-corrected cyst volume and cyst index fraction. One study [23] assessed peripheral blood mononuclear cell (PBMC) phosphorylated-AMPK (p-AMPK) to AMPK (p-AMPK/AMPK). Finally, a change in liver function was evaluated through cyst volume and fraction and height-adjusted total liver volume (htTLV). From a clinical prespective, several trials prioritized feasibility and metabolic endpoints, whereas renal structural endpoints were often assessed over durations that are short, relevant to the typical pace of ADPKD progression. This is relevant when interpreting « unchanged » findings, because typically, structural progression is slow and small changes may not be detected in short-term follow up.

### 3.5 Intervention adherence

Not all studies assessed adherence to the allocated interventions. In further detail, Hopp [23] and Ekinci [29] did not mention ketosis assessment, whereas the rest of the studies included either fingertip blood, urine pH or used breath analyzers.

### 3.6 Quality of included studies

**Fig. 3:**
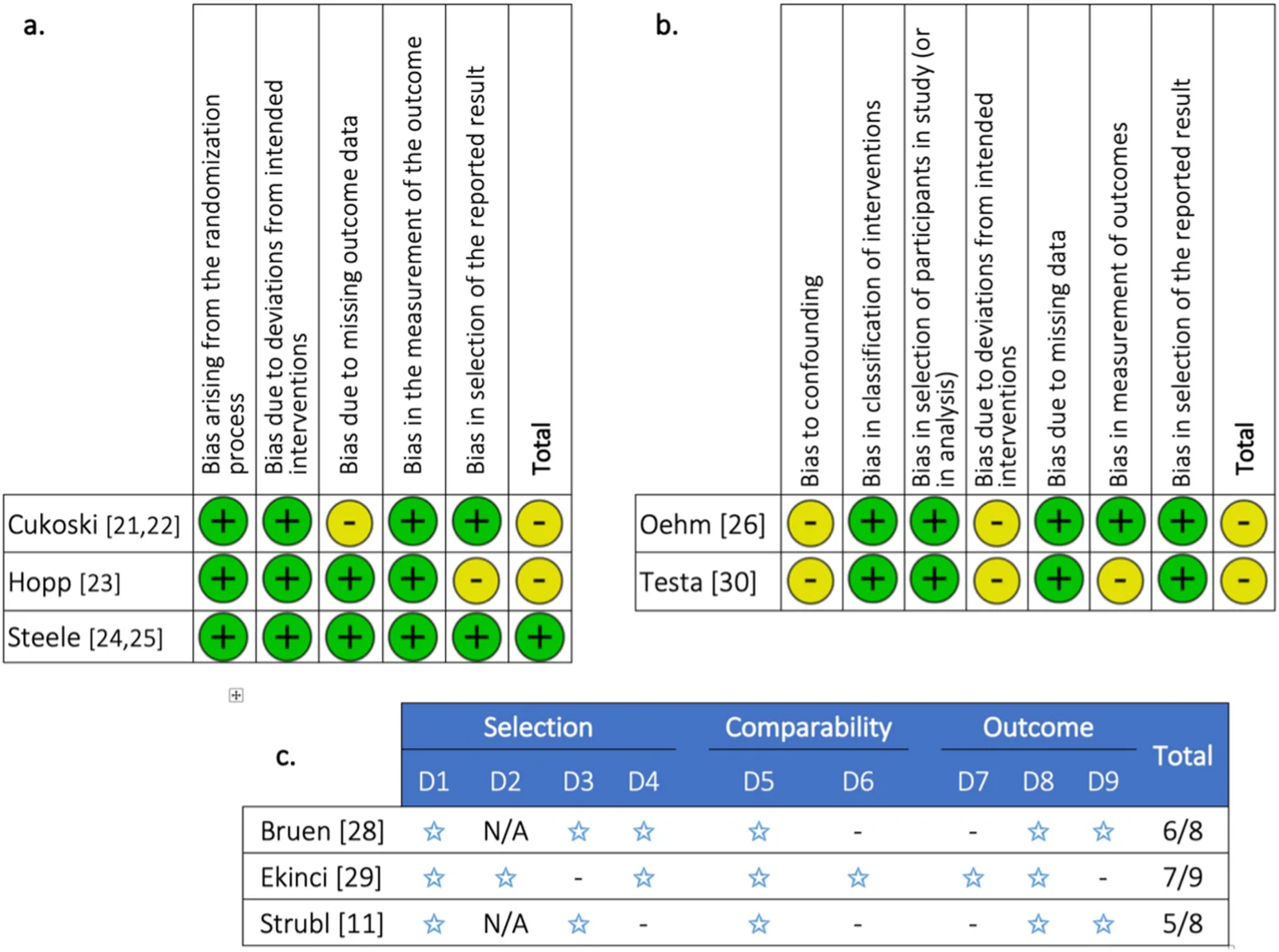
Risk of bias of the included studies. 3a. Risk of bias in randomized controlled trials using the RoB2 tool. 3b. Risk of bias in unrandomized trials using the Robins II tool. 3c. Risk of bias in cohort studies utilizing the Newcastle-Ottawa scale.

All included studies were of moderate to good quality. In RCTs, issues arose due to the unavoidable awareness of the intervention by participants. The Steele trial [24,25] demonstrated the less RoB from all included RCTs. With regard to unrandomized clinical trials, most of the identified bias involved confounding and deviations from the intented interventions. In cohort studies, the main issue of concern involved loss of follow-up. Additional details can be found in Fig. 3.

### 3.7 SWiM of the results

**Fig. 4:**
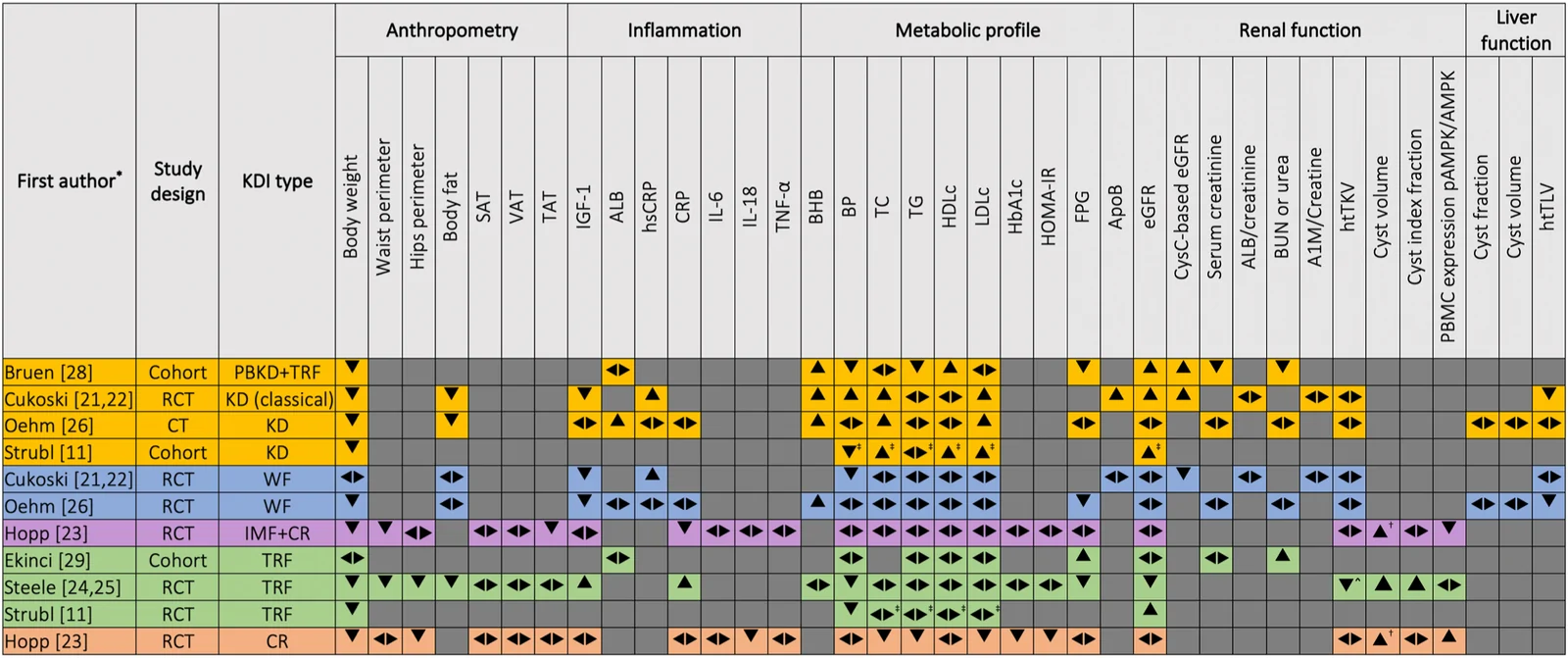
SWiM (qualitative synthesis) of the results of studies assessing the efficacy of KDIs in patients with ADPKD. A1M: Alpha-1-microglobulin; ADPKD, autosomal dominant polycystic kidney disease; ALB: albumin; AMPK: AMP-activated protein kinase; ApoB: apolipoprotein B; BHB: beta-hydroxybutyrate; BP: blood pressure; BUN: blood urea nitrogen; CRP: C-reactive pro-tein; CysC: systatin C; CT: clinical trial; FPG: fasting plasma glucose; eGFR: estimated glomerular filtration rate; HbA1c: glycosylated haemoglobin; HDLc: high density lipoprotein cholesterol; HOMA-IR: Homeostatic Model Assessment for Insulin Resistance; hsCRP: high-sensitivity C-reactive protein; htTKV: height adjusted TKV; IGF-1: insulin-growth factor 1; IMF: intermittent fasting; IL-6: interleukin 6; IL-18: interleukin 18; KD: ketogenic diet; KDI: ketogenic dietary inter-vention; LDLc: low-density lipoprotein cholesterol; NR: not reported; pAMPK: phosphorylated AMP-activated protein kinase; PBKD: plant-based ketogenic diet; PCMC: peripheral blood mon-onuclear cell; RCT: randomized controlled trial; SAT: subcutaneous adipose tissue; TAT: total adipose tissue; TC: total cholesterol; TG: triglycerides; TKV: total kidney volume; TLV: total liver volume; TNF-α: tumor necrosis factor α; TRF: time-restricted feeding; VAT: visceral adipose tis-sue; WF: water fasting. * Testa [30] provided individual patient changes instead of average sam-ple results; ‡ self-reported. † height corrected.

The SWiM of the results trend using vote counting is detailed in Fig. 4. Testa [30] provided individual patient data without group values, so the study was omitted from the SWiM. Fig. 4 shows the direction of effects across studies, however vote counting does not convey effect size or clinical importance, while statistically non-significant findings may reflect limited power rather than true absence of effect.

For interpretability, outcomes are summarized below as (i) anthropometry/metabolic effects and (ii) renal disease modifying endpoints. Fig. 4 includes comparisons on the direction of effects.

#### 3.7.1 Anthropometry and metabolic effects

Overall, KDIs were associated with BW loss and an improved anthropometric profile in most studies (9 interventions), irrespective of the selected dietary pattern. Waist and hips circumferences (2 interventions) and body fat (%BW, 3 interventions) were also reduced when vote counting was applied. SAT, VAT and TAT appeared unaffected.

In most research, IGF-1, CRP and hsCRP levels remained similar post-intervention, alongside IL-6, IL-18 and TNF-α. BHB concentrations were improved post-intervention, indicating a more “ketogenic” metabolism.

Arterial BP remained unchanged in most interventions (five) [21,23,26,29], whereas four interventions revealed a reduction in BP [11,24,25,28] (in one, it was self-reported), while Cukoski [21,22] showed a non-significant increase trend in the BP of participants undergoing KD and a non-significant trend towards a BP decrease in the WF group. TC remained unchanged in most of the interventions [11,[21], [22], [23], [24], [25], [26],^28^], revealing a decline in one (CR) [23] and an upward trend in three interventions [11,21,22,26]. TG concentrations mostly remained unchanged [11,[21], [22], [23],^26^,^29^] or trended downwards in two interventions [23,28]. HDLc was improved in two interventions [11,28] but remained unchanged in most treatment arms [[21], [22], [23],^26^,^29^]. As for LDLc, it noted a decrease in one intervention [23], an increase in three others [11,21,22,26] and remained unaffected in the rest of the studies [[21], [22], [23],^26^,^28^,^29^]. FPG was either decreased or remained akin to pre-intervention concentrations. Notably, ketosis and body weight loss did not fully overlap as reportable oucomes, because several studies did not quantify ketone exposure. Therefore, while weight loss was a consistent finding across interventions, the degree of ketosis cannot be inferred uniformly across studies.

Overall these findings support metabolic feasibility and benefits of KDIs, especially regarding anthropometry and weight loss.

#### 3.7.2 Renal outcomes

Concerning renal outcomes, eGFR was either higher post-intervention (4 interventions) [11,21,22,28] or remained stable [[21], [22], [23],^26^,^29^]. Cystatin C (CysC)-based eGFR was mostly improved when KDs were followed [21,22,28] but a decrease was noted when patients adhered to WF [21,22]. htTKV remained unchanged in most of the studies [[21], [22], [23],^26^] with Steele [24,25] reporting a reduced rate of increase among participants in the KDI compared to controls. Additionally, Cukoski [21,22] reported a decline in htTKV that was not statistically significant; however patients achieving high biochemical thresholds of ketosis significantly decreased htTKV compared to controls. However, no study was designed to demonstrate sustained slowing of htTKV growth over the long term, which is the key structiral marker used to infer disease modification in ADPKD. Three interventions [[23], [24], [25]] reported increased height-corrected cyst volume, although cyst index fraction remained similar to pre-intervention values. [23]. Finally, PBMC expression pAMPK/AMPK, the ratio of PBMC protein expression of p-AMPK/AMPK, remained unchanged when vote counting was applied. Concerning liver outcomes, cyst fraction and volume remained unchanged [21,22,26] whereas htTLV was either reduced [21,22,26] or similar to pre-intervention concentrations [21,22,26]. It is important to note that most interventions were short in duration and not powered to detect long-term renal progression. Therefore, short-term changes or stability in kidney function should not be interpreted as slowing down eGFR decline.

## 4 Discussion

The present systematic review showed that research on the KDIs in ADPKD is limited, with many research protocols still ongoing. In parallel, KDIs are feasible in ADPKD and, based on the limited available research, evidence is more consistent for metabolic benefits than for proven renal disease modifications. Some disease-specific outcomes have been reported to change. For example, eGFR and CysC-based eGFR may be ameliorated following KDIs in the short term, whereas htTKV seems to remain unchanged, and htTLV is either reduced or similar to pre-intervention volumes. Importantrly, current studies do not provide definite evidence that KDIs slow htTKV or slow long term eGFR decline. Renal outcomes were heterogenous and largely derived from short-duration interventions.

As far as metabolic outcomes are concerned, KDIs seem to induce favorable results regarding body weight and the anthropometric profile of patients, whereas inflammation and blood lipids, in the majority, remain unaffected. The overall level of evidence can be interpreted as follows: evidence is most consistent for short-term metabolic effects, whereas evidence is currently insufficient to conclude renal disease modification.

Recently, Cukoski [21] noted that the Warburg effect is apparent in ADPKD, with mitochondrial dysfunction, glutamine repletion, and changes in the synthesis and oxidation of tricarboxylic and fatty acids [36,37]. KDIs have been hypothesized to act by suppressing the mTOR pathway, while activating the liver kinase B1/AMP-activated protein kinase pathway [38]. Research in mice showed that a moderate food restriction (10%–40%) was associated with slowed down course of the disease, reducing inflammation, cyst area, renal fibrosis, and injury in a ketone dose-dependent manner [8,38]. Similarly, renal cystic disease progression was also shown in mice on the TRF diet [27]. On the other hand, research on patients with ADPKD [39] revealed that glucagon was negatively associated with eGFR and htTKV. In contrast, BHB concentrations were negatively associated with disease severity and slower renal function decline [12]. These results suggest a possible therapeutic pathway by tampering down circulating glucagon concentrations and increasing BHB concentrations via the induction of ketosis. In parallel, due to the slow and progressive nature of the disease, while the primary goal of most therapies is to halt disease progression [8], emerging evidence raises the possibility that partial disease reversal may be achievable.

Due to the restrictive nature of KDIs, long-term adherence is difficult to achieve. However, for some conditions (i.e., drug-resistant epilepsy), it may be a one-way solution for life. According to Strubl [11], 53% of the patients reported breaks during their diet, indicating that long-term adherence may be hard to achieve. Ketogenic dietary patterns are generally high in fat and low in carbohydrates, with varying protein content [10]. As a result, the occasional consumption of higher protein content by mistake may be possible, if patients are inadequately trained, making KDIs a dietary pattern that most healthcare professionals may be reluctant to recommend for safety precautions. In general, deviations from the typical ADPKD dietary recommendations [40,41] associated with greater protein intake might induce unfavorable effects on the patients’ renal health. However, the increasing information regarding the role of KDIs in ADPKD indicates that KDIs are feasible and may also be effective and risk-free when monitored closely. However, education on the KDIs is required for healthcare professionals who prescribe them to patients.

WF is another way to achieve ketosis in less time and at a higher degree than following a KD [42]. The present synthesis showed that for many of the available research items, WF was prescribed as a control intervention [21,22,26]. The results (two RCTs) revealed that for most of the examined parameters, WF was not consistently associated with changes in the inflammatory, metabolic or ADPKD-related outcomes compared to interventions with KDs. On a side note, however, we must acknowledge that not all outcomes were similar in the two RCTs and that WF cannot be a feasible dietary pattern followed for a long period, as it is associated with increased hunger, a negative energy balance and possibly, micronutrient deficiencies.

As per Cukoski [21], another concern regarding the adoption of KDIs is a potentially negative result on the cardiovascular risk of patients, which is an important issue, particularly for renal disease. Indeed, in the experience of people with ADPKD with KDs cohort [11], most patients reported concerns regarding possible hyperlipidemia following KDIs. The present synthesis showed that following KDIs, BP is most likely reduced (or remains unchanged). However, the only negative finding regarding the blood lipid profile was the possible increase in LDLc and TC, as noted by three interventions [21,22,26]. Cukoski [21,22] showed that adherence to the KD was associated with greater LDLc and very LDL (VLDL) size, phospholipid content in LDL, free cholesterol, cholesteryl esters, sphingomyelins, and the ApoB/ApoA1 ratio at the end of the interventions. When adhering to KDs, a decrease in small LDL particles occurs [43]. In contrast, as patients with obesity are concerned [44] although LDLc may be increased in KDIs, it is the proportion of large-sized buoyant LDL (with cardioprotective effects) that is increased and not the more atherogenic one (small-sized dense LDL). In patients with ADPKD, a factor for the increased LDLs levels may be the dietary content of saturated fatty acids (SFA), with potential food sources being cheese, butter, meat, and even coconut oil [21]. In parallel, lean individuals tend to experience the most marked increase in LDLc concentrations, known as the lean hyper-responder phenotype [21,45].

As seen in the present systematic review, KDIs are highly heterogeneous; nonetheless, research indicates that their adoption is associated with improved body weight and composition when excess body weight is apparent [[46], [47], [48]]. A key interpretive issue is whether reported improvements are attributable to ketosis itself, or are secondary to body weight reduction and improved metabolic status. Hopp et al. [23] showed that a change in htTKV was related to the percent change in body weight. TKV was not only lower among participants who had lost a clinically significant amount of body mass compared to the rest, but also showed a trend toward reduction in this group, suggesting a posible signal of reduced cystic burden. Therefore, this reported relationship between renal volumetric outcomes and changes in body weight tends to support the role of weiglt-loss mediated pathways. At the same time, Cukoski [21,22] described that when patients were stratified by ketosis level, those reaching high biochemical levels of ketosis exhibited reduced htTKV compared to controls. This suggests that greater ketosis thresholds may be associated with more favorable renal trajectories. These findings are further supported by observational data from the DIPAK cohort, where higher BHB concentrations were accosiated with a slower decline in kidney function [12]. In parallel, according to animal research, a reduced renal weight of animals on CR was associated with a decrease in cystic index, number, and size, suggesting a signal but not proving renal disease modification [23]. These observations suggest that the reduction in body weight observed when on KDIs might, in fact, drive improvements in ADPKD-related outcomes [23]. However, ketosis and body weight loss are tightly coupled in most KDIs and not all studies quantify ketone exposure. Although a decrease in TKV was not apparent in all studies in contrast to TLV, it is hypothesized that TKV may require longer interventions compared to TLV [26]. Additionally, a significant body weight loss might further aid in the reduction of TKV [23]. Therefore, disentangling weight-loss mediated effects, from ketosis specific effects is critical for future studies, particularly when interpreting renal endpoints. Future research on the effect of KDIs on ADPKD should continue to evaluate lipid concentrations to aid understanding of the exact results driven by KDIs.

At the moment, the literature is still young on the use of KDIs for ADKPD, with many animal studies and few human trials. This is why the latest recommendations for nutrition in ADPKD and current guideline statements (KDIGO) [49] emphasize a balanced, healthy diet and weight management and indicate that there is currently insufficient evidence to recomment any specific diet as disease-modifying care in ADPKD. Accordingly, KDIs cannot yet be recommented routinely for ADPKD in clinical practice and should be considered, if at all, within supervised clinical trials or highly individualized settings, with close multidisciplinary monitoring. Nonetheless, new research is underway [[31], [32], [33]] to better delineate the exact effect of KDIs or refute their efficacy. KDIs are restrictive dietary patterns that need commitment from the patients’ side and experience from the side of healthcare professionals. It is also important to design studies comparing the typical healthy eating recommendations for ADKPD (standard care) [40,41] to KDIs in a longitudinal manner, in order to help us understand the effects of the diet better, with renal endpoints framed as disease-modifying outcomes rather than short-term changes in kidney function markers. Furthermore, it would be interesting to see if and how often patients deviate from the prescribed protein intake by exceeding it, when on KDIs.

The present systematic review included a qualitative synthesis but not a meta-analysis due to the heterogeneity of the included studies in terms of interventions, comparators and study design. Furthermore, this heterogeneity did not allow for the grading of the recommendations according to the GRADE. In addition, the available human evidence is limited by small sample sizes in several trials, including small proof-of-principle and uncontrolled studies. In the future, when the results of the ongoing studies are published, a quantitative synthesis will be feasible and less heterogeneous for certain outcomes. Another limitation involves using different KDIs (WF, IMF, TRF, CR) as controls, thus comparing two KDIs in most of the available research. This design limits interpretability, because it makes it difficult to attribute effects to ketosis versus caloric deficit or time-restriction, and it does not reflect the real clinical question of whether KDIs provide benefits in renal outcomes. A proper design would have compared KDIs to the standard of care diet for ADPKD, namely the dietary recommendations for this condition [40,41]. An additional key limitation is the short duration of most interventions and limited follow-up for renal outcomes. Given the slow progression of ADPKD, short interventions are unlikely to detect meaningful changes in disease-modifying endpoints such as sustained slowing of htTKV or long term eGFR decline. Intervention heterogenity was also substantial, with variability in KDI type, macronutrient targets, ketosis assessment methods and daily calories, which may contribute to inconsistent findings across studies and limits generalizability. Finally, not all the studies included herein excluded patients on Tolvaptan. As this could have affected the results, it is advised to rely on the results of RCTs rather than observational cohorts, where treatment is not controlled.

## 5 Conclusions

The evidence on KDIs for ADPKD is still limited and current studies provide more consistent support for short term metabolic feasibility than for proven renal disease modification, particularly regarding sustained slowing of htTKV growth and long-term eGFR decline. Nevertheless, KDIs, particularly approaches incorporating restricting calories may represent a promising non-pharmatological adjunct for selected patients with ADPKD. KDIs should be delivered with appropriate supervision and safety monitoring. From a public health perspective, dietary strategies that safely improve weight status and cardiometabolic risk factors could have meaningful impacts in ADPKD. However, KDIs are restrictive and may carry risks (eg dyslipidemia in some individuals, renal stones, micronutrient inadequacy and reduced adherence), underscoring the need for structured education, monitoring and access to specialist support. Future research should prioritize longitudinal trials that compare KDIs to standard of care dietary recommendations, are designed to distinguish ketosis-specific effects from weight loss effects and determine which subgroups are more likely to benefit. Importantly, more research is required to understand the exact effect of KDIs on ADPKD, synthesize data, and recommend or refute adherence to specific KDIs.

## CRediT authorship contribution statement

**Maria G. Grammatikopoulou:** Writing – review & editing, Writing – original draft, Visualization, Supervision, Methodology, Investigation, Data curation, Conceptualization. **Arriana Gkouvi:** Writing – review & editing, Writing – original draft, Visualization, Methodology, Investigation, Data curation. **Kalliopi K. Gkouskou:** Writing – review & editing. **Dimitrios Poulimeneas:** Writing – review & editing, Investigation. **Christina Tsigalou:** Writing – review & editing. **Τheodoros Eleftheriadis:** Writing – review & editing. **Odysseas Androutsos:** Writing – review & editing. **Christos Cholevas:** Writing – review & editing. **Ioannis Stefanidis:** Writing – review & editing. **Maria Dalamaga:** Writing – review & editing. **Dimitrios G. Goulis:** Writing – review & editing, Methodology, Conceptualization. **Dimitrios P. Bogdanos:** Writing – review & editing, Supervision, Data curation.

## Data availability statement

As this is a meta-research item, all created data are available on this manuscript and its supplementary material.

## Funding

This work did not receive funding.

## Declaration of competing interest

Given her role as co-Editor-in-chief, Prof Maria Dalamaga had no involvement in the peer review of this article and had no access to information regarding its peer review. Full responsibility for the editorial process regarding this article was delegated to another journal editor. The rest of the authors, declare that they have no known competing financial interests or personal relationships that could have appeared to influence the work reported in this paper.

## List of abbreviations:

A1M: α-1-microglobulin
Ac: Acetone
ADPKD: Autosomal Dominant Polycystic Kidney Disease
AMPK: AMP-activated protein kinase
ApoB: Apolipoprotein β
BHB: Beta-hydroxybutyrate
BP: Blood pressure
BUN: Blood-urea nitrogen
BW: Body weight
CENTRAL: Cochrane Central Register of Controlled Trials
CKD: Chronic kidney disease
CR: Caloric restriction
CRP: C-reactive protein
CysC: Cystatin C
ERK: Extracellular signal-regulated kinase
ESKD: End-stage kidney disease
FPG: Fasting plasma glucose
HbA_1c_: Glycated hemoglobin
HDLc: High-density lipoprotein cholesterol
Hs-CRP: High sensitivity CRP
htTKV: Height-adjusted total kidney volume
htTLV: Height-adjusted total liver volume
IGF-1: Insulin-growth factor-1
IL: Interleukin
IMF: Intermittent fasting
KDI: Ketogenic dietary interventions
KD: Ketogenic diets
LDLc: Low-density lipoprotein cholesterol
MAD: Modified Atkins diet
MCT: Medium-chain triglycerides
NOS: Newcastle-Ottawa scale
ONS: Oral nutrient supplements
OSF: Open Science Framework
p-AMPK: Phosphorylated-AMPK
PBMC: Peripheral blood mononuclear cell
PICOS: Population, Intervention, Comparison, Outcomes, Study design
PRISMA: Preferred Reporting Items for Systematic reviews and Meta-Analyses
QoL: Quality of life
RCT: Randomized controlled trial
RoB: Risk of Bias
ROBINS-II: Risk Of Bias In Non-randomized Studies of Interventions
SAT: Subcutaneous adipose tissue
SFA: Saturated fatty acids
SWiM: Synthesis WIthout Meta-analysis
TAT: Total adipose tissue
TC: Total cholesterol
TG: Triglycerides
TKV: Total kidney volume
TNF-α: Tumor-necrosis factor α
TRF: Time-restricted feeding
VAT: Visceral adipose tissue
VLDL: Very low density lipoprotein
WF: Water fasting
