# Supplementary material for: Ketogenic dietary interventions for autosomal-dominant polycystic kidney disease (ADPKD): a systematic review and synthesis without meta-analysis (SWiM) of observational and interventional studies
Source: Metabol Open. 2026 Jan 31;29:100447. doi: 10.1016/j.metop.2026.100447 (PMC12907129; doi:10.1016/j.metop.2026.100447)
Supplement: Multimedia component 1 [file mmc1.docx]

Supplementary material

**Supplementary Table 1.** PICOS of the study’s research question.

| **P**opulation | Patients with ADPKD |
| --- | --- |
| **I**ntervention | KDI, including KD, TRF, CR |
| **C**omparison | Any comparison, including usual diet, other KDI patterns |
| **O**utcomes | Any PKD-related outcome, including renal function, BP, eGFR, |
| **S**tudy design | Any design with comparator arms, including case-control, pre-post studies, non-randomized CT, RCT |

ADPKD, autosomal dominant polycystic kidney disease; BP, blood pressure; CR, caloric restriction; CT, clinical trial; eGFR, estimated glomerular filtration rate; KD, ketogenic diet; KDI, ketogenic dietary interventions; RCT, randomized controlled trial; TRF, time-restricted feeding.

**Supplementary Table 2.** List of excluded studies.

| First author | Title | Exclusion rationale |
| --- | --- | --- |
| Afsar, 2021 | The effect of energy restriction on development and progression of chronic kidney disease: review of the current evidence | Review |
| Amro, 2016 | Low-Osmolar Diet and Adjusted Water Intake for Vasopressin Reduction in Autosomal Dominant Polycystic Kidney Disease: A Pilot Randomized Controlled Trial | Irrelevant results |
| Arnold, 2017 | Randomized, Controlled Trial of the Effect of Dietary Potassium Restriction on Nerve Function in CKD | Irrelevant study |
| Best, 2023 | Effects of very low carbohydrate ketogenic diets on skeletal health | Review, irrelevant results |
| Boletta, 2016 | Slowing Polycystic Kidney Disease by Fasting | Commentary on included article |
| Bolignano, 2015 | Interventions for preventing the progression of autosomal dominant polycystic kidney disease | Review |
| Bruci, 2020 | Very Low-Calorie Ketogenic Diet: A Safe and Effective Tool for Weight Loss in Patients with Obesity and Mild Kidney Failure | Different population |
| Capelli, 2023 | Diet and Physical Activity in Adult Dominant Polycystic Kidney Disease: A Review of the Literature | Review |
| Carney, 2020 | Ketosis slows the progression of PKD | Review |
| Carriazo, 2019 | Dietary Care for ADPKD Patients: Current Status and Future Directions | Review |
| Chang, 2022 | Wang's Yijing Decoction Combined with Conventional Therapy: performance in Treating Polycystic Ovary Syndrome with Kidney Deficiency and Phlegm-dampness Syndrome and Regulating Effect on Intestine-brain Axis | foreign language |
| Chebib, 2023 | Polycystic Kidney Disease Diet: What is Known and What is Safe. | Review |
| ChiCTR2100054284 | A clinical trial of ketogenic diet in the treatment of autosomal dominant polycystic kidney disease | Registry, foreign language |
| Conley, 2021 | Interventions for weight loss in people with chronic kidney disease who are overweight or obese | Review |
| Cukoski, 2024 | Ketosis moderates the effect on kidney volume in dietary interventions for ADPKD-more insights on the KETO ADPKD trial | Conference abstract of included article |
| Dachy, 2022 | Is autosomal dominant polycystic kidney disease an early sweet disease? | Review |
| Kashyap, 2020 | Implications of the PAPP-A-IGFBP-IGF-1 pathway in the pathogenesis and treatment of polycystic kidney disease | Review |
| Kędzierska-Kapuza, 2024 | The Influence of Non-Pharmacological and Pharmacological Interventions on the Course of Autosomal Dominant Polycystic Kidney Disease | Review |
| Kipp, 2016 | A mild reduction of food intake slows disease progression in an orthologous mouse model of polycystic kidney disease | Animal study |
| Kocyigit, 2020 | Association of OSR-1 With Vascular Dysfunction and Hypertension in Polycystic Kidney Disease | Non ketogenic, irrelevant study |
| Li, 2020 | Ketogenic diets and protective mechanisms in epilepsy, metabolic disorders, cancer, neuronal loss, and muscle and nerve degeneration | Review |
| Liu, 2023 | Ketone Body Metabolism and Renal Diseases | Foreign language |
| Mahendran, 2021 | Natural-derived compounds and their mechanisms in potential autosomal dominant polycystic kidney disease (ADPKD) treatment | Review |
| Meijer, 2021 | Emerging non-pharmacological interventions in ADPKD: an update on dietary advices for clinical practice | Review |
| Messchendorp, 2018 | Association of plasma somatostatin with disease severity and progression in patients with autosomal dominant polycystic kidney disease | Non ketogenic, irrelevant study |
| Messing, 2024 | Trigger Warning: How Modern Diet, Lifestyle, and Environment Pull the Trigger on Autosomal Dominant Polycystic Kidney Disease Progression | Review |
| Michalski, 1996 | The effect of hypervolemia on electrolyte level and and level of volume regulating hormones in patients with autosomal dominant polycystic kidney disease | foreign language |
| Mutaal, 2024 | Exploring the role of ketogenic diet in ADPKD treatment: A promising avenue | Letter describing included studies |
| NCT01233869 | Bosutinib For Autosomal Dominant Polycystic Kidney Disease | Irrelevant study |
| NCT02497521 | The German ADPKD Tolvaptan Treatment Registry (AD(H)PKD) | Irrelevant study |
| NCT02964273 | Safety, Pharmacokinetics, Tolerability and Efficacy of Tolvaptan in Children and Adolescents With ADPKD (Autosomal Dominant Polycystic Kidney Disease | Irrelevant study |
| NCT03342742 | {Daily Caloric Restriction and Intermittent Fasting in Overweight and Obese Adults With Autosomal Dominant Polycystic Kidney Disease} | Registry of included study, Hopp et al |
| NCT03687554 | Effect of Venglustat in Patients With Renal Impairment | Irrelevant study |
| NCT04407481 | PErfusioN, OxyGen ConsUmptIon and ENergetics in ADPKD (PENGUIN) (PENGUIN) | Irrelevant study |
| NCT04472624 | Short Term Induction of Ketosis in PKD (RESET-PKD) | Registry of included study, RESET-PKD |
| NCT04534985 | Time Restricted Feeding in Autosomal Dominant Polycystic Kidney Disease | Registry of included study, Steele et al |
| NCT04680780 | Ketogenic Dietary Interventions in Autosomal Dominant Polycystic Kidney Disease (ADPKD) (Keto-ADPKD) | Registry of included study, Keto-ADPKD |
| NCT04907799 | Daily Caloric Restriction in ADPKD | Not finished |
| NCT06100133 (ADKETONE) | Treat Autosomal Dominant Polycystic Kidney Disease With Oral Ketone Ester? (ADKETONE) | Not finished |
| NCT06325644 | Well-Formulated Ketogenic Diet Polycystic Kidney Disease | Not finished, recruiting |
| NCT06496542 (EXPLORE) | Renal Oxygen Consumption, Insulin Sensitivity, and Daily Caloric Restriction in ADPKD | Not finished, recruiting |
| Nowak, 2020 | Metabolic Reprogramming in Autosomal Dominant Polycystic Kidney Disease: Evidence and Therapeutic Potential | Review |
| Nowak, 2021 | Weight loss to slow cyst growth in autosomal dominant polycystic kidney disease (ADPKD) | Abstract of included study |
| Ong, 2022 | Can ketogenic dietary interventions slow disease progression in ADPKD: what we know and what we don't | Editorial comment |
| Palmer, 2017 | Dietary interventions for adults with chronic kidney disease | Review |
| Pezzuoli, 2023 | GREASE II: A PHASE II RANDOMIZED, 24-MONTH, PARALLEL-GROUP, SUPERIORITY STUDY TO EVALUATE THE EFFICACY OF A KETOGENIC DIET IN ADPKD PATIENTS | Abstract, study not finished |
| Pezzuoli, 2024 | Ketogenic Interventions in Autosomal Dominant Polycystic Kidney Disease: A Comprehensive Review of Current Evidence | Review |
| Pickel, 2022 | Dietary Interventions in Autosomal Dominant Polycystic Kidney Disease | Review |
| Pierre, 2024 | Interventions for preventing the progression of autosomal dominant polycystic kidney disease | Review |
| Pietrzak-Nowacka, 2015 | Association of kidney and cysts dimensions with anthropometric and biochemical parameters in patients with ADPKD | Non ketogenic, irrelevant results |
| Rosati, 2024 | Potential Add-On Benefits of Dietary Intervention in the Treatment of Autosomal Dominant Polycystic Kidney Disease | Review |
| Singh, 2019 | Dietary restriction regimens for fighting kidney disease: Insights from rodent studies. | Review |
| Steele, 2021 | Metabolomic changes over 1 year following drug or lifestyle interventions in autosomal dominant polycystic kidney disease (ADPKD) | Metabolomics study, irrelevant results |
| Testa, 2020 | GREASE II. A phase II randomized, 12-month, parallel-group, superiority study to evaluate the efficacy of a Modified Atkins Diet in Autosomal Dominant Polycystic Kidney Disease patients | Study protocol |
| Torres, 1994 | Renal cystic disease and ammoniagenesis in Han:SPRD rats | Animal study |
| Torres, 2019 | Ketosis Ameliorates Renal Cyst Growth in Polycystic Kidney Disease | Animal study |
| Torres, 2023 | A Combination of ß-Hydroxybutyrate and Citrate Ameliorates Disease Progression in a Rat Model of Polycystic Kidney Disease. | Animal study |
| Torres, 2024 | β-hydroxybutyrate recapitulates the beneficial effects of ketogenic metabolic therapy in polycystic kidney disease | Animal study |
| Wang, 2024 | Changes in tubular biomarkers with dietary intervention and metformin in patients with autosomal dominant polycystic kidney disease: a post-hoc analysis of two clinical trials | post hoc analysis |
| Warner, 2016 | Food Restriction Ameliorates the Development of Polycystic Kidney Disease | Animal study |
| Weimbs, 2024 | Ketogenic metabolic therapy for chronic kidney disease - the pro part | Review |

**Supplementary Table 3**. Inclusion and exclusion criteria of the included studies.

| **First author** | **Inclusion and exclusion criteria** |
| --- | --- |
| Bruen | **Inclusion:** ADPKD diagnosis, age 18 yr or older, eGFR ≥ 30 mL/min/1.73 m^2^. Active treatment with tolvaptan did not preclude enrollment.  **Exclusion:** DM type 1, active eating disorder, kidney transplant, hyperkalemia, metabolic instability, malabsorption issues, pregnancy, and eGFR < 30 mL/min/1.73 m^2^. |
| Cukoski | Inclusion: CKD stage G1-3 AND at least one indicator of rapid disease progression (Mayo class 1C-E OR truncating *PKD1* mutation OR onset of arterial HT or urological symptoms <35 yr OR 1st- or 2nd-degree family members reaching kidney failure at <60 yr OR eGFR loss >2.5 mL/min/year OR PROPKD score >6).  **Exclusion:** Underweight or obese, exposure to a KD > 2 wks within the last 6 mo, participation in a weight-loss program within the last 6 mo, vegan, current treatment (or within the last 6 mo) with tolvaptan or somatostatin analog, conditions prohibiting the use of a KD, eating disorders, alcohol abuse, DM type 1, insulin-dependent DM type 2, contraindications to MRI, pregnancy or breastfeeding, absence of safe contraceptive measures or non-occurrence of menopause. |
| Ekinci | **Inclusion:** patients over 18 yr with ADPKD.  **Exclusion:** patients on renal replacement therapy, history of kidney transplant, type-1 or type-2 DM, uncontrolled or poorly-controlled HT, AKI, acute tubular necrosis due to dehydration, history of colic pain attacks due to nephrolithiasis/urolithiasis, malignancy, active infection, chronic liver disease, advanced cardiac disease, diabetes insipidus, inflammatory diseases, pregnancy, or active peptic ulcer. |
| Hopp | **Exclusion:** DM (diagnosis, fasting glucose >126 mg/dL or HbA1C > 6.5%), current nicotine use or history of use in the past 12 mo, alcohol or substance abuse (self-report or undergoing treatment), history of hospitalization or major surgery within the last 3 mo, untreated dyslipidemia, uncontrolled HT (SBP >160 mmHg or DBP >100 mmHg). Pregnant, lactating, or unwilling to use adequate birth control women. History of cardiovascular disease, peripheral vascular disease, cerebrovascular disease, significant pulmonary disorders (including COPD, ILD, cystic fibrosis, or uncontrolled asthma), significant gastrointestinal disease (including chronic malabsorptive conditions, peptic ulcer disease, Crohn’s disease, ulcerative colitis, chronic diarrhea, or active gallbladder disease), cancer (within the last five yr, except for skin cancer or other cancers considered cured with excellent prognosis), or untreated hypo- or hyperthyroidism (TSH outside of normal range for laboratory or history of uncontrolled thyroid disorder; history of thyroid disorder or current thyroid disease treated with stable medication regimen for at least 6 mo was acceptable). Abnormal resting ECG (serious arrhythmias, including multifocal PVCs, frequent PVCs (defined as >10/min), ventricular tachycardia (runs of 3 or more successive PVCs), or sustained atrial tachyarrhythmia; 2nd or 3rd degree A-V block, QTc interval > 480 msec or other significant conduction defects), regular use of prescription or over-the- counter medications that may affect weight, appetite, food intake, or energy metabolism (e.g., appetite suppressants, lithium, stimulants) or regular use of obesity pharmacotherapeutic agents within the last 6 mo, weight loss >5% in the past 3 mo for any reason except post-partum weight loss, weight gain >5% in the past 3 mo, or history of a clinically diagnosed eating disorder including anorexia nervosa, bulimia [binge eating disorder score >20 on the Eating Attitudes Test (EATS-26)] (Garner et al., 1982) required further assessment by the Study MD. Current severe depression or history of severe depression within the previous yr, based on DSM-IV-TR criteria for Major Depressive Episode or score >18 on the Beck Depression Inventory (Beck et al., 1988) required further assessment by the Study MD. Finally, participants were excluded if they had a history of other significant psychiatric illness (psychosis, schizophrenia, mania, bipolar disorder) which in the opinion of the Study MD would interfere with their ability to adhere to dietary interventions. Individuals with an inability to cooperate with/clinical contraindication for MRI including severe claustrophobia, implants, devices, or non-removable body piercings were not included in this procedure but could still enroll in the study. Prevalent tolvaptan usage was not exclusionary, although no participants that enrolled were using tolvaptan. |
| Oehm | **Inclusion:** ADPKD patients aged 18–60 yr with an eGFR ≥45 mL/min/1.73 m^2^ and rapidly progressing disease defined as the presence of at least one of the following criteria: Mayo Class 1C–1E, truncating *PKD1* mutation, early onset of HT (before 35 yr), early onset of urological complications (before 35 years of age), historical eGFR decline >2.5/year or a PROPKD score >6.  **Exclusion:** current tolvaptan therapy, BMI <18 or >35 kg/m^2^, DM, alcohol addiction, vegan or vegetarian, known circumstances prohibiting the use of a KD {e.g. liver damage, pyruvate carboxylase deficiency, defects of gluconeogenesis, defects of ketolysis/ketoneogenesis, hyperinsulinism, defects of fatty acid oxidation}, allergies and intolerances to components of a KD, eating disorders, participation in a weight loss program or taking weight loss promoting medication in the last 6 mo, KD > 1 mo in the last 12 mo, chronic renal replacement therapy, previous kidney transplantation, pregnancy or contraindications to MRI scans. |
| Steele | **Inclusion:** Age 18–65 yr, ADPKD diagnosis based on the modified Pei-Ravine criteria, BMI 25–45 kg/m^2^ CKD-EPI eGFR ≥30 mL/min/1.73 m^2^ access to the internet with video chat capabilities and smartphone, typical eating duration >12 hrs/day, not currently participating in another interventional study or weight loss program, ability to provide informed consent.  **Exclusion:** DM (diagnosis or fasting glucose >126 mg/dL or HbA1C >6.5%), current nicotine use or history of use in the past 12 mo, alcohol or substance abuse (self-report or undergoing treatment), history of hospitalization or major surgery within the last 3 mo, untreated dyslipidemia, uncontrolled HT (SBP > 160 or DBP > 100 mmHg), pregnancy, lactation, or unwillingness to use adequate birth control, cardiovascular disease, peripheral vascular disease, cerebrovascular disease, significant pulmonary disease, cancer (within the last 5 yr, except skin cancer or other cancers considered cured with excellent prognosis), significant gastrointestinal disorders including: chronic malabsorptive conditions, peptic ulcer disease, Crohn's disease, ulcerative colitis, chronic diarrhea, or active gallbladder disease, regular use of prescription or over-the-counter medications that may affect weight, appetite, food intake, or energy metabolism (e.g. appetite suppressants, lithium, stimulants, anti-psychotics, tricyclic antidepressants; study physician will be consulted as needed; antibiotics started during the intervention period are not an exclusion); regular use of obesity pharmacotherapeutic agents within the last 6 mo, history of clinically diagnosed eating disorder including anorexia nervosa, bulimia, binge eating disorder. Score >20 on the Eating Attitudes Test (EATS)-2653 will require further assessment by the Study MD. Weight loss >5% in past 3 mo for any reason except post-partum weight loss; weight gain >5% in past 3 mo requires further assessment. Untreated hyper- or hyperthyroidism (TSH outside of normal range for laboratory or history of uncontrolled thyroid disorder, history of thyroid disorder or current thyroid disease treated with stable medication regimen for at least 6 mo is acceptable). Current severe depression or history of severe depression within the previous yr, based on DSM-IV-TR criteria for Major Depressive Episode. Score > 18 on the Beck Depression Inventory will require further assessment by the Study MD. History of other significant psychiatric illness (e.g. psychosis, schizophrenia, mania, bipolar disorder) which in the opinion of the Study MD would interfere with ability to adhere to dietary interventions. Inability to cooperate with/clinical contraindication for MRI including severe claustrophobia, implants, devices, or non-removable body piercings |
| Strubl | **Inclusion:** ADPKD patients who tried self- initiated KDIs in the past. KDIs include variations of KDs, TRDs and CR.  **Exclusion:** Participants on dialysis, with kidney transplants or on dietary protocols that were not ‘KDI conformable’. |
| Testa | **Inclusion:** Men and women that were eligible to Tolvaptan treatment, according to the Italian Drug Agency criteria of reimbursability, but declined the treatment, mainly because of the aquaretic activity of the drug. In brief, patients were aged 18–50 yr, with a renal function comprised in the range 45–89 ml/min/1.73 m^2^ according to the CKD-EPI formula, with large kidneys (according to US or MRI scan) and evidence of rapidly progressive disease.  **Exclusion:** DM type I and II, gastro- intestinal malabsorption problems, eating behavior disorders (anorexia, bulimia). |

ADPKD: autosomal-dominant polycystic kidney disease; BMI: body mass index; CKD: Chronic kidney disease; COPD: chronic obstructive pulmonary disease; DBP: Diastolic blood pressure; DM: Diabetes mellitus; ECG: Electrocardiogram; eGFR: estimated glomerular filtration rate; HbA1C: glycated haemoglobin; HT: Hypertension; ILD: interstitial lung disease; KD: Ketogenic diet; MD: Medical doctor; MRI: Magnetic resonance imaging; mo: month; PKD1: Polycystin 1; PROPKD: Predicting Renal Outcome in Polycystic Kidney Disease; PVC: Premature ventricular contraction; SBP: Systolic blood pressure; TSH: thyroid-stimulating hormone; US: ultrasound; wk: week; yr: years.
